# Effectiveness of ophthalmic solution preservatives: a comparison of latanoprost with 0.02% benzalkonium chloride and travoprost with the sofZia preservative system

**DOI:** 10.1186/1471-2415-11-8

**Published:** 2011-04-21

**Authors:** Gerard Ryan, Joel M Fain, Cherie Lovelace, Karl M Gelotte

**Affiliations:** 1 Development Analytics, Pfizer Inc, Eastern Point Road, Groton, CT, USA; 2 Pfizer Specialty Care, 3453 North Racine Avenue, Chicago, IL, USA; 3 Independent Research Consultant, Kennesaw, GA, USA; 4 Life Cycle Management, Pharmaceutical Development, Pfizer Inc, Groton, CT, USA

## Abstract

**Background:**

Although in vitro and in vivo laboratory studies have suggested that benzalkonium chloride (BAK) in topical ophthalmic solutions may be detrimental to corneal epithelial cells, multiple short- and long-term clinical studies have provided evidence supporting the safety of BAK. Despite the conflicting evidence, BAK is the most commonly used preservative in ophthalmic products largely due to its proven antimicrobial efficacy. This study was designed to characterize the antimicrobial performance of two commonly used topical ocular hypotensive agents that employ different preservative systems: latanoprost 0.005% with 0.02% BAK and travoprost 0.004% with sofZia, a proprietary ionic buffer system.

**Methods:**

Each product was tested for antimicrobial effectiveness by *European Pharmacopoeia *A (EP-A) standards, the most stringent standards of the three major compendia, which specify two early sampling time points (6 and 24 hours) not required by the *United States Pharmacopeia *or *Japanese Pharmacopoeia*. Aliquots were inoculated with between 10^5 ^and 10^6 ^colony-forming units of the test organisms: *Staphylococcus aureus, Pseudomonas aeruginosa, Escherichia coli, Candida albicans *and *Aspergillus brasiliensis*. Sampling and enumeration were conducted at protocol-defined time points through 28 days.

**Results:**

BAK-containing latanoprost met EP-A criteria by immediately reducing all bacterial challenge organisms to the test sensitivity and fungal challenges within the first six hours while the preservative activity of travoprost with sofZia did not. Complete bacterial reduction by travoprost with sofZia was not shown until seven days into the test, and fungal reduction never exceeded the requisite 2 logs during the 28-day test. Travoprost with sofZia also did not meet EP-B criteria due to its limited effectiveness against *Staphylococcus aureus*. Both products satisfied United States and Japanese pharmacopoeial criteria.

**Conclusions:**

Latanoprost with 0.02% BAK exhibited more effective microbial protection than travoprost with sofZia using rates of microbial reduction, time to no recovery for all challenges and evaluation against EP-A criteria as measures. The rapid and complete reduction of all microbial challenges demonstrates that antimicrobial activity of latanoprost with 0.02% BAK exceeds that of travoprost with sofZia preservative system in these products and provides a more protective environment in the event of contamination and subsequent exposure to microorganisms during use.

## Background

Adequate preservation is of paramount importance in ophthalmic solutions packaged in multidose containers to minimize the risk of infection associated with inadvertent microbial contamination. Yet, even when preserved with benzalkonium chloride (BAK), microbial contamination has been found to be present in 28% to 29% of in-use containers [1,2], with a significantly greater frequency in those used for more than 8 weeks [2]. This contamination translated into a high concordance of the same organisms cultured from the conjunctiva [1,2], especially in patients with ocular surface disease (OSD) [1]; one-third of patients reported having touched their eyes during medication installation [1]. Coagulase-negative *Staphylococcus*, *Staphylococcus aureus *and a variety of gram-negative bacteria that are not usual conjunctival flora were among the potentially pathogenic organisms identified [1,2]. In more recent studies using video recordings to evaluate the performance of patients with ocular hypertension or glaucoma, supported by Alcon, only a third of patients were actually successful at instilling a single drop of medication without touching the eye or ocular adnexae [3,4].

A quaternary ammonium compound with bacteriostatic and bacteriocidal properties, BAK has been used to preserve ophthalmic medications since the late 1940s [5]. Today, more than 70% of ophthalmic medications available in multidose containers, including topical ocular hypotensive agents, contain BAK in concentrations typically ranging from 0.004% to 0.02% [6]. An alternative preservative system, sofZia^®^, an ionic buffer that contains borate, sorbitol, propylene glycol and zinc [7], recently has been developed and approved by the U.S. Food and Drug Administration. SofZia has been used since as an alternative to BAK (0.015%) in Travatan Z^® ^(Alcon, Inc. Fort Worth, Texas, USA), another available formulation of travoprost ophthalmic solution.

The present study characterizes and compares the antimicrobial performance of these preservatives in the commonly used topical ocular hypotensive agents latanoprost 0.005% with 0.02% BAK [8] and travoprost 0.004% with sofZia [7]. Both products are approved for sale in the United States [7,8] and Japan [9], and latanoprost ophthalmic solution is approved in the European Union (EU). While travoprost ophthalmic solution with 0.014% BAK is approved in the EU [10], travoprost with sofZia is not.

Standards for preservative effectiveness are set forth in the *European Pharmacopoeia *(EP) [11], including both EP-A and EP-B criteria for antimicrobial activity, and in the *United States Pharmacopeia *(USP) [12] and the *Japanese Pharmacopoeia *(JP) [13]. These recognized standards were used as comparative assessment measures in this work. The EP (edition 6.6, chapter 5.1.3) [11] test for efficacy of antimicrobial preservation of ophthalmic preparations using the EP-A evaluation criteria for antimicrobial activity is widely recognized for evaluating preservative effectiveness in pharmaceutical products marketed in EP member states. The EP-A evaluation demands two early sampling time points (6 and 24 hours) not required by either the USP (chapter 51) [12] or the JP (general information chapter 19) [13], therefore representing the most stringent of the three major compendia. The EP-B criteria are reserved for justified cases where criteria A cannot be attained, such as products that would be of "increased risk of adverse events."

## Methods

The antimicrobial effectiveness testing was conducted at Lancaster Laboratories (Lancaster, Pennsylvania, USA), an independent laboratory, according to the EP-A standards (EP-A; Tables 1 and 2[11]). Additional measurement time points and microorganisms were included to allow for the evaluation of results against EP evaluation criteria B (EP-B; Table 2) and the USP [12] and the JP [13] standards (Table 1). Latanoprost ophthalmic solution 0.005% with 0.02% BAK or travoprost 0.004% ophthalmic solution with sofZia were tested against the following bacteria and fungi: *Staphylococcus aureus *(ATCC 6538)*, Pseudomonas aeruginosa *(ATCC 9027), *Escherichia coli *(ATCC 8739), *Candida albicans *(ATCC 10231) and *Aspergillus brasiliensis *(a subspecies of *Aspergillus niger*; ATCC 16404). These organisms were selected based on EP [11] and USP [12] test protocols. According to the standard methodology, the bulk dilution was split into 10 mL aliquots, which were inoculated with between 10^5 ^and 10^6 ^colony-forming units (CFU)/mL of each organism (1 organism per aliquot) and stored at 20°C to 25°C. Sampling and enumeration of the inoculated samples were done at protocol-defined time points through 28 days (Table 1) [11]. One mL aliquots were serially diluted in typtone-azolectin-Tween broth and plated in duplicate on tryptic-soy agar (for bacteria) or Sabouraud dextrose agar (for fungi). Plates were incubated at 30°C to 35°C for ≥3 days for bacteria and 20°C to 25°C for ≥5 days for fungi. Raw data counts were converted to log_10 _values and the reduction from inoculum values was calculated for evaluation against compendial requirements. Since the samples were diluted at least 1:10 at the time of testing, 10 CFU (or 1.0 log reduction) is the lowest sensitivity allowed by the test.

**Table 1:** Microorganisms and postinoculation time points tested in this protocol and required by EP-A and USP/JP [11-13]

Microorganisms included in all tests			
*Pseudomonas aeruginosa*			
*Staphylococcus aureus*			
*Candida albicans*			
*Aspergillus brasiliensis*			
Escherichia coli*			

**Time points**	**Protocol**	**EP-A**	**USP/JP**

0 hour	T	T	T
6 hours	T	T	N
24 hours	T	T	N
7 days	T	T (mold only)	T
14 days	T	N	T
28 days	T	T	T

JP: Japanese Pharmacopoeia; N: not included in test; T: included in test; USP: United States Pharmacopeia.

*By European Pharmacopoeia-A (EP-A) standards, only required for oral products [11].

**Table 2:** Parenteral and ophthalmic preparation European Pharmacopoeia criteria A and B (With permission from the European Pharmacopoeia[11])

	Log reduction					
		**6 hours**	**24 hours**	**7 days**	**14 days**	**28 days**
	
Bacteria	A	2	3	-	-	NR
	B	-	1	3	-	NI

Fungi	A	-	-	2	-	NI
	B	-	-	-	1	NI

NI: no increase; NR: no recovery.

The recovery methods of the enumeration procedures were qualified by comparing the recovery of representative microorganisms (at a low concentration of ≤100 CFU) from the test article to the recovery from positive controls. At 1:10, all bacteria and fungi had a recovery rate of ≥95% (1:10 dilution) and ≥87% (1:100 dilution), respectively, which is within the 70% to 200% range demonstrating suitability of the recovery method (data not shown).

The primary endpoints were the differences between latanoprost and travoprost ophthalmic solutions in their alignment with the EP-A criteria A, the time to "no recovery" (report of <10 CFU or <1.0 log) for each organism/product combination and the recovered organism counts at 6 and 24 hours as defined in EP standards.

## Results

**Figure 1:**
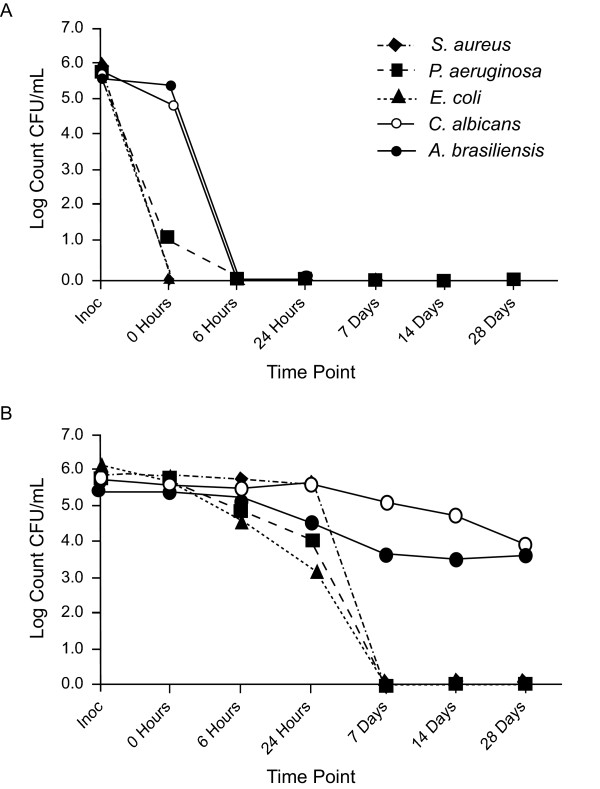
**Reduction in microorganism counts over 28 days with (A) latanoprost with benzalkonium chloride (BAK) and (B) travoprost with sofZia. CFU: colony-forming unit; Inoc: inoculation.** For ease of graphing <1.0 log was plotted as "0".

Latanoprost ophthalmic solution 0.005% with 0.02% BAK exceeded EP-A criteria with reductions of all bacterial challenge microorganisms (≥4.7 log at 0 hours) and all fungal challenge microorganisms (≥4.4 log at 6 hours) (Table 3). These results exceeded the requisite 2 log reductions for bacteria at 6 hours and 2 log reductions for fungi at 7 days. Travoprost with sofZia did not meet the EP-A criteria, demonstrating a mean reduction of only 0.5 log (range: 0.1, 1.5) in bacterial counts at 6 hours. At 24 hours, the mean bacterial reduction for travoprost was 1.1 log (range: 0.1, 2.8); reductions ≥4.7 log did not occur until day 7. The fungal counts never exceeded the requisite reductions (2 logs at 7 days) for the duration of the 28-day test (Table 3; Figures 1A and 1B).

**Table 3:** Microbial reduction (log_10 _CFU/mL) by time point

Sample	Microorganism	Inoculum	0 hours	6 hours	24 hours	7 days	14 days	28 days
Latanoprost 0.005% (Lot No: LA54019)	*S. aureus*	5.8	> 4.8	> 4.8	> 4.8	> 4.8	> 4.8	> 4.8
	*P. aeruginosa*	5.7	4.7	> 4.7	> 4.7	> 4.7	> 4.7	> 4.7
	*E. coli*	6.0	> 5.0	> 5.0	> 5.0	> 5.0	> 5.0	> 5.0
	*C. albicans*	5.7	0.9	> 4.7	> 4.7	> 4.7	> 4.7	> 4.7
	*A. brasiliensis*	5.4	0.1	> 4.4	> 4.7	> 4.4	> 4.4	> 4.4
Travoprost 0.004% (Lot No: 158568F)	*S. aureus*	5.8	0	**0.1**	**0.2**	> 4.8	> 4.8	> 4.8
	*P. aeruginosa*	5.7	0.1	**0.8**	**1.7**	> 4.7	> 4.7	> 4.7
	*E. coli*	6.0	0.3	**1.5**	**2.8**	> 5.0	> 5.0	> 5.0
	*C. albicans*	5.7	0.1	0.2	0.1	**0.6**	1.0	1.8
	*A. brasiliensis*	5.4	0.1	0.2	0.9	**1.7**	1.9	1.8

CFU: colony-forming unit.

Latanoprost ophthalmic solution 0.005% contains 0.02% benzalkonium chloride and travoprost ophthalmic solution 0.004% contains sofZia as preservatives.

Shading indicates results not meeting European Pharmacopoeia-A (EP-A) requirements [11] (note that while testing of *E. coli *is required only for non-oral products in the EP, this would be considered a failing result).

Since travoprost did not meet EP-A criteria, the results were evaluated against EP-B criteria, which require reductions in bacterial counts of 1 and 3 logs at 24 hours and 7 days, respectively, with no increase at 28 days, and a 1 log reduction in fungal counts at 14 days, with no increase at 28 days. These less stringent criteria are reserved for products for which suitable justification exists for not meeting EP-A criteria, such as an increased risk of adverse reactions [11]. When evaluated against EP-B criteria (Table 2), travoprost still did not satisfy EP requirements due to its limited effectiveness against *Staphylococcus aureus *at 24 hours (Table 3). There was the required 1 and 3 log reductions for *Pseudomonas aeruginosa *and *Escherichia coli *at 24 hours and 7 days, respectively. However, while travoprost marginally satisfied EP-B criteria for fungi at 14 days (1.0 and 1.9 log reductions for *Candida albicans *and *Aspergillis brasiliensis*, respectively), reductions were far less than those achieved at 6 hours by latanoprost with 0.02% BAK.

## Discussion

When compared with the compendial requirements [11], BAK-containing latanoprost exceeded the EP-A criteria at all time points [11]. Travoprost with sofZia, however, while meeting USP standards [6,14], did not meet the EP-A criteria for either bacteria or fungi, exhibiting only modest reductions at 6 and 24 hours, nor did it meet EP-B criteria due to its limited effectiveness against *Staphylococcus aureus*. *Staphylococcus *infections are frequently associated with both primary and recurrent bleb infections following trabeculectomy [15] and endophthalmitis subsequent to postoperative procedures such as lens replacement [16]. *Pseudomonas aeruginosa *is also a common cause of endophthalmitis, occurring postoperatively or subsequent to corneal ulcers, and is often associated with poor visual outcomes [17].

The early time points, which assess the rate of kill of the challenge organisms, revealed the most significant differences between the two preservative systems. Latanoprost with 0.02% BAK exhibited complete reduction of a large microbial insult (bacterial and fungal) within the first 6 hours of exposure while travoprost with sofZia showed only modest reductions. These results are especially important as the early time points simulate microbial contamination that may occur upon use and be present over the next 24 hours after use. In addition, the fungal/yeast challenge never reached a point of "no recovery" in the travoprost samples during the 28-day test.

Benzalkonium chloride also has been shown to be more effective than other preservatives when measured against the EP-A criteria [11]. When artificial tears containing BAK (0.01%)/ethylenediaminetetraacetic acid (EDTA; 0.05%), chlorobutanol (0.5%), stabilized oxychloro complex (50 parts per million), sodium silver chloride complex (0.001%) or methyl-, ethyl - and propylparaben (undeclared concentration) were compared [18], the product containing BAK/EDTA alone satisfied the criteria for all test microorganisms. The majority of products failed the criteria for one or more bacteria, notably with the 6- and 24-hour samples. An agar diffusion test also was performed, with only the BAK/EDTA sample showing a zone of inhibition; the effect was shown to be due to BAK only since other products without EDTA gave similar results [18].

Recent studies in which patients were videotaped to assess their success at instillation of topical ocular hypotensive medications highlight the concerns about bottle contamination [3,4]. In the first of these studies, 92.8% of patients with a diagnosis of glaucoma or ocular hypertension who used 1 or more glaucoma medications for at least 6 months reported no problems administering their eye medications; yet, less than a third of patients were successful at instilling a single drop with touching the bottle to the eye [3]. In a subsequent study in patients with visual impairment or moderate to severe visual field loss, only 39% were able to instill a single drop without touching the eye; age (<70 vs ≥70 years) was found to be a significant predictor for less successful instillation [4]. These studies demonstrate that bottle contamination is a more important issue than previously believed.

There has been an ongoing controversy about the contributions of BAK-containing ophthalmic solutions to ocular toxicity, particularly using in vitro studies and rabbit models, many with exaggerated-use protocols (for reviews, see Kaur et al [6] and Furrer et al [19]). The relevance of these studies to the clinical setting is not well established given the various methodologies, models, exposure times and concentrations. Several recent studies, all sponsored by Alcon, have compared travoprost to latanoprost or bimatoprost with respect to ocular tolerability in glaucoma patients [20-23]. In the first of these studies, patients (n = 691) who required alternate therapy due to tolerability issues were switched from either latanoprost or bimatoprost to travoprost with sofZia [20]. While there was no significant difference in the reported OSD index (OSDI) scores between patients who were classified as normal at baseline (n = 456), patients who were symptomatic at baseline (n = 235) reported significant improvements in their scores 3 months after switching to travoprost. However, as the authors indicated, the study design (nonrandomized, nonmasked) was limited so that expectation of improvement may have resulted in patients subjectively reporting a more favorable outcome.

In other studies involving patients with preexisting OSD, tolerability findings have been inconsistent. In one double-blind study, patients who were receiving latanoprost and reported ocular dryness and irritation (n = 33) were randomized to receive latanoprost in one eye and travoprost with sofZia in the other eye; eyes were assessed by a single examiner every 3 to 4 weeks for 3 months, and patients completed an OSDI survey [23]. Significant increases in corneal staining occurred in the travoprost-treated eyes compared to the latanoprost-treated eyes, with OSDI surveys also showing a trend toward more dryness and irritation symptoms in the travoprost eyes. There were no differences in tear breakup times (TBUT), intraocular pressure, visual acuity or Schirmer testing between the two groups [23]. In an open-label, prospective study of patients (n = 20) with a baseline TBUT of less than 6 seconds, significant increases in mean TBUT and decreases in mean OSDI scores and corneal staining were reported 8 weeks after switching from latanoprost to travoprost with sofZia [21].

In contrast, in a prospective, double-masked, randomized comparative study of 54 subjects, there were no significant differences between latanoprost and travoprost with sofZia with respect to reported discomfort scores following a single instillation of either agent [22]. In a small, prospective, observational cohort study with masked examiners supported by Merck [24], patients with glaucoma or ocular hypertension (naive to treatment, n = 10; previously on latanoprost, n = 8) were instructed to use latanoprost in the right eye and travoprost with sofZia in the left eye. There was statistically significantly less conjunctival hyperemia in eyes treated with latanoprost (both in the naive and previously treated patients) and in corneal staining in eyes previously treated with latanoprost but no statistical difference in TBUT, change in intraocular pressure from baseline, or impression cytology between the treatment groups [24]. None of these studies specifically assessed the incidence of ocular infections or rates of bottle contamination; additional studies are warranted.

Patients receiving ocular hypotensive medications are reported to have a high prevalence of OSD, with 59% of patients reporting OSDI symptoms in at least one eye; Schirmer testing was abnormal in 61% of patients and TBUT was decreased in 78% of patients [25]. After adjustment for age and sex, factors considered to influence the prevalence of OSD, multivariate logistic regression found that the use of BAK-containing agents was associated with a two-fold increase risk of lissamine green staining in the 22% of patients with positive results (none had severe staining based on a scale of 0 to I, normal; II to III, mild to moderate; and IV to V, severe). These rates are higher than those reported in population-based studies, which likely reflects the fact that some of these patients may have been referred due to OSD symptoms or may have been treated with multiple preservative-containing eye drops [25]. This hypothesis is consistent with the results of a retrospective analysis of three large prescription databases sponsored by Pfizer [26]. Patients newly treated with latanoprost or travoprost with sofZia and without a diagnosis of dry eye or ocular infection in the prior 6 months had no significant differences in the rates of dry eye or ocular infections (identified by International Classification of Diseases, Ninth Revision, Clinical Manifestation code or by prescription for cyclosporine ophthalmic emulsion or ocular antibiotics) at 1 year [26].

Of importance in considering the findings of the present study, patients with OSD have an increased risk of microbial keratitis [27-29], with OSD found to be a predisposing factor in 21% of cases of bacterial keratitis in one study [27] and in 15% of case in another study [28]. *Staphylococcus *species were found to be the most commonly isolated organisms in OSD-associated bacterial keratitis [27,29]. Moreover, a history of OSD was found to be significantly associated with a "very poor" visual outcome following bacterial keratitis [23]. Thus, the failure of travoprost to satisfy even EP-B requirements due to the limited effectiveness against *Staphylococcus aureus *at 24 hours raises concerns about the adequacy of its preservation.

To date, in clinical usage and in observational studies, the findings are mixed with regard to ocular tolerability and may depend upon the study design. While some switch studies found improvements in tolerability when switching from bimatoprost/latanoprost to travoprost with sofZia [20,21], one study found increases in corneal staining and irritation when switching from travoprost with sofZia to latanoprost [23]. Still, in the large, retrospective study sponsored by Pfizer [26], there were no significant differences in dry eye or infection between patients receiving latanoprost versus travoprost with sofZia. The randomized, masked, clinical registration study sponsored by Alcon comparing travoprost with BAK to travoprost with sofZia found no differences in adverse events or safety endpoints [30]. Therefore, the presumed benefits of BAK-free or other alternative preservative systems in terms of ocular tolerability remain to be clearly established.

## Conclusion

The rapid microbial reduction, along with the complete reduction of all microbial challenges with latanoprost ophthalmic solution with BAK, demonstrates that its antimicrobial activity exceeds that of travoprost with the sofZia preservative system and will afford greater protection against contamination and subsequent exposure to microbial insults during normal use. This antimicrobial activity is reassuring in the typical patient with glaucoma who is predisposed to OSD.

## Competing interests

GR, Jr, JMF, CL, and KMG are employees of Pfizer Inc and own Pfizer Inc stock and stock options. The research was funded by Pfizer Inc.

## Authors' contributions

GR, Jr, JMF, and KMG are employees of Pfizer Inc and own Pfizer Inc stock and stock options. CL was an employee of Pfizer Inc at the time the study was conducted. The research was funded by Pfizer Inc

## Pre-publication history

The pre-publication history for this paper can be accessed here:

http://www.biomedcentral.com/1471-2415/11/8/prepub
